# *Colletotrichum gloeosporioides*- Contaminated Tea Infusion Blocks Lipids Reduction and Induces Kidney Damage in Mice

**DOI:** 10.3389/fmicb.2017.02089

**Published:** 2017-10-30

**Authors:** Jin Li, Kang Sun, Qingping Ma, Jin Chen, Le Wang, Dingjun Yang, Xuan Chen, Xinghui Li

**Affiliations:** ^1^Tea Research Institute, Nanjing Agricultural University, Nanjing, China; ^2^Institute of Soil & Fertilizer and Resources & Environment, Jiangxi Academy of Agricultural Science, Nanchang, China

**Keywords:** *Colletotrichum gloeosporioides*, food contamination, green tea, health-care function, renal damage

## Abstract

When the homogenate of fresh tea tree leaves was fermented to produce black tea beverage, the *Colletotrichum gloeosporioides* (main pathogen or endophyte of *Camellia sinensis*) may be mixed into the fermentation liquor. However, it was unclear whether *C. gloeosporioides-*contaminated tea beverage would damage human health. Therefore, we investigated the changes of functional components and the influences on mice. *C. gloeosporioides* was added to the green tea infusion. After cultivation of 48 h, tea polyphenols, caffeine, and L-theanine decreased by 31.0, 26.2, and 8.3%, respectively. The contaminated tea infusion showed brown stain, and produced a group of toxic materials named phthalic acid esters. The animal study showed that green tea without contamination significantly decreased levels of alanine aminotransferase, triglycerides, free fatty acids, low-density lipoprotein, and increased insulin level compared with obese mice. On the contrary, contaminated tea lost the effects on these indicators. Furthermore, the urea nitrogen and serum creatinine levels significantly increased in the contaminated tea-drinking mice. Altogether, our results indicate that *C. gloeosporioides* contamination can reduce the amount of functional components of green tea. Therefore, it inhibits some health-care function of lipid-lowering. In addition, the toxic components in contaminated tea infusion might induce renal damage.

## Introduction

Tea (*Camellia sinensis*) is one of the major alcohol-free beverages in the world and very popular for its health benefits, which include reduction of lipids, regulation of blood sugar levels, prevention of cardio-cerebrovascular diseases, and protection of liver (Bolling et al., 2009; Fon et al., 2011). The main functional components of tea include tea polyphenols, caffeine, and L-theanine. The leaves of tea tree are the raw materials for the production of tea. As a result, the quality of tea leaves could affect the contents of functional components in the tea products, thereby influencing the activities of health-care functions.

*Colletotrichum gloeosporioides*, is considered as the world’s eighth largest plant pathogen (Dean et al., 2015) and widely distributed in mango, banana, carica papaya, strawberry, coffee, cocoa, onion, potato, and other tropical and subtropical crops. It causes anthracnose in plants which results in falling of leaves, deterioration of fruits and vegetables, as well as adverse effects on the yield and quality of agricultural products (Cannon et al., 2012). *C. gloeosporioides* is also one of the major pathogen of tea plants in worldwide (Copes and Thomson, 2008; Liu et al., 2015). However, C. *gloeosporioides* remains as parasite for a long time in tea leaves and stems as a non-pathogenic endophyte state (Rabha et al., 2016), making it difficult to distinguish the infected leaves from uninfected raw fresh tea leaves. Therefore, it is not clear whether it will impact on the quality of tea beverage products and food safety.

In this study, *C. gloeosporioides* fungus was added to the extracted green tea infusion and then determined the functional components of tea infusion. Subsequently, the lipid reduction effect of green tea on C57BL/6J high-fat-fed mice was investigated, followed by verification using ICR mice. We aimed to reveal the influence of *C. gloeosporioides* on health-care functions and safety of tea.

## Materials and Methods

### *C. gloeosporioides* Isolation and Identification

**FIGURE 1:**
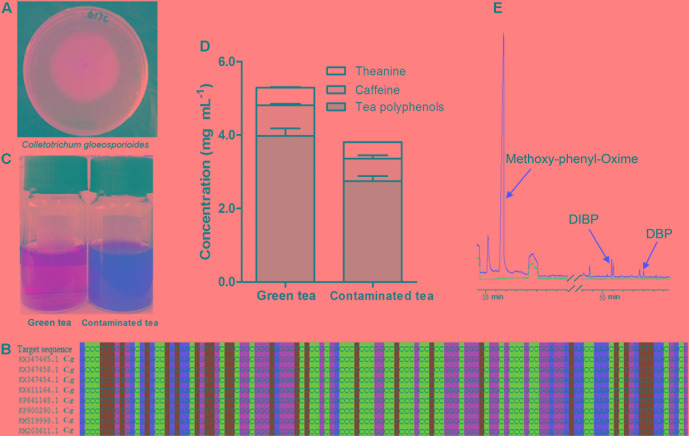
*Colletotrichum gloeosporioides* and the influence on green tea infusion. The colony shape of *C. gloeosporioides* **(A)** and comparison of nucleotide sequences in ITS region on NCBI **(B)**. Optical image of green tea and *C. gloeosporioides*-contaminated tea **(C)**. Changes of functional components contents **(D)**. GC/MS chromatograms of green tea (blue) and contaminated tea (red) **(E)**. Mean + SEM.

After surface sterilization with 75% ethanol for 2 min and 0.5% sodium hypochlorite for 2 min, tea leaves were cut into 50 mm × 50 mm pieces, and placed in the potato dextrose agar (PDA) medium surface. Then tea leaves were cultivated at 28°C for 48 h. Pure cultures were obtained by single spore isolation and maintained on PDA plates at 28°C for 7 days, before observation of cultural and morphological characteristics. The cultures of *C. gloeosporioides* exhibited white, gray, and orange color, while the reverse side of the colonies was of white and dark gray (**Figure 1A**). Then, the pure hyphae were collected and the DNA extracted to measure the ITS of nuclear ribosomal DNA to identify the fungi species (Stirling, 2003). The primer sequences were ITS1: 5′-TCCGTAGGTGAACCTGCGG-3′ and ITS4: 5′-TCCTCCGCTTATTGATATGC-3′. Nucleotide sequences of sequenced ITS region were aligned using BLAST in NCBI^1^. Nucleotide sequences of ITS region of all isolates had 100% homology with *C. gloeosporioides* (GenBank: KX347465.1) isolates available in the NCBI (**Figure 1B**).

### Preparation of Tea Infusion

Fresh tea leaves were collected from Suchazao, a variety of the tea plant (*C. sinensis*), and then, produced into green tea after de-enzyming and torrefaction. To prepare green tea infusion, 2 g of green tea was mixed with 100 mL distilled water, and extracted at 100°C for 30 min. Tea infusion was collected, and sterilized at 120°C for 20 min. Stored strains (PDA slants at 4°C) were activated twice on PDA medium at 28°C for 2 days before each inoculation. Then fresh mycelium was inoculated into sterilized tea infusion. Later the green tea and mycelium-additive green tea extractions were cultured at 28°C for 48 h, the changes of the color and contents of the mycelium additive extractions were no longer noticeable. So the culture conditions were selected for the follow-up test.

### Detection of Components in Tea Infusion

The color of tea infusion before and after fermentation was observed by Chroma meter, CR-400 colorimeter (Osaka, Japan) (Leon et al., 2006). Color was expressed as L^∗^, a^∗^, and b^∗^, which indicate luminosity, chromaticity on a green (-) to red (+) axis, and chromaticity on a blue (-) to yellow (+) axis, respectively (Chen et al., 2010). The volatile components in the tea infusion were detected by GC–MS method (Bruker 450GC and 320MS, Karlsruhe, Germany). Briefly, 10 mL extracted tea infusion and 10 mL contaminated tea infusion were preheated at 70°C for 10 min in 15 mL of headspace bottle, respectively. The solution was extracted at 50°C for 60 min with magnetic stirring by using Solid Phase Micro Extraction (Supelco Inc., Bellefonte, PA, United States). GC conditions were: inlet temperature 250°C, detector temperature 250°C, high purity helium, flow rate 1 mL/min, initial column temperature 40°C for 5 min, elevated (3°C/min) to 210°C for 5 min, then elevated (10°C/min) to 250°C for 5 min. MS conditions were: interface temperature 280°C, inlet temperature 250°C, ion source temperature 230°C, electronic energy 70 eV, quadrupole temperature 150°C, ionization mode EI and scanning range 35–600 a.m.u. Chemical compositions were identified by searching NIST database. Moreover, spectrophotometry was used to detect the total content of polyphenols (GB/T 8313-2008), and HPLC (Alliance 2695, Waters, Milford, MA, United States) was adopted to detect the contents of caffeine (GB/T 8312-2013) and L-theanine (GB/T 23193-2008).

### Animals and Animal Treatments

Healthy male C57BL/6J mice (body weight of 10–12 g, 3–4 weeks) were purchased from Laboratory Animal Centre of Yangzhou University (Permission number: 201604129), China and high-fat diet (60% fat calories) and paired regular diet (10% fat calories) for feeding C57BL/6J mice were purchased from TROPHIC Animal Feed High-Tech Co. Ltd., China. Healthy male ICR mice (18–20 g, 6–7 weeks) and their diet were purchased from Changzhou Cavens Laboratory Animal Co., Ltd. (Permission number: 201606808), China. The mice were housed in plastic cages in the Laboratory Animal Centre of Nanjing Agricultural University with controlled temperature (20–26°C), humidity (40–70%), light intensity 250 lx, and 14:10 h light:dark cycle. This study was carried out in accordance with the recommendations of the guidelines of American Association for Accreditation of Laboratory Animal Care. The protocol was approved by the Animal Use and Care Committee of Nanjing Agricultural University.

Twenty-eight C57BL/6J male mice were randomized into four groups (*n* = 7). LF group were fed with low-fat diet and clear water, and HF group were fed with high-fat diet and clear water. HF + GT group was given high-fat diet and green tea, and HF + CT group was given high-fat diet and *C. gloeosporioides*-contaminated tea infusion. All animals had free access to diet and drinking for 8 weeks. The amounts of drinking water and feed, as well as change of body weight were recorded daily. At the end of this experiment, mice were held without feed for 12 h before bleeding. In the second morning serum were collected from the tail blood for determination of fasting blood glucose (FBG).

Twenty-four normal ICR mice were randomized into three groups (*n* = 8) and fed with normal diet for 2 weeks: CK group was given clear water, GT group was given green tea, and CT group was given *C. gloeosporioides-*contaminated tea infusion.

### Tissue Preparation and Biomarker Assessments

At the end of each experiment, mice were fasted for 12 h, anesthetized, and sacrificed by cervical dislocation after peripheral blood collection from the ophthalmic vein. Serum was obtained by centrifugation at 10,000 rpm for 10 min, and stored at -80°C until analysis. Liver, kidney, and small intestine tissues were excised and rinsed in ice-cold saline and then stored at -80°C.

For histological studies, the liver, kidney, and small intestine were obtained from treated and control C57BL/6J mice. Tissues were fixed by 4% paraformaldehyde in 0.01 M PBS for 24 h, and then processed for paraffin inclusion. Serial paraffin-sections (4 mm) were stained with hematoxylin–eosin (HE) followed by light microscopy examination (KingMed Diagnostics, Nanjing, China).

Alanine aminotransferase (NJJC-C009), aspartate aminotransferase (AST) (NJJC-C010), S-Cr (NJJC-C011), and BUN (NJJC-C013) in serum were determined by spectrophotometry using commercially available kits according to the manufacturer’s instructions. Serum contents of monocyte chemoattractant protein-1 (MCP-1) (RGB&CHN-60224M), TNF-α (AngleGene-E21030M), LPS (AngleGene-E21618M), and INS (RGB&CHN-60207M) were detected using ELISA Kit according to the manufacturer’s instructions.

Triglyceride (TG) (NJJC-A110), TC (NJJC-A111), LDL (NJJC-A113), HDL (NJJC-A112), FFA (NJJC-A045), and methane dicarboxylic aldehyde (MDA) (NJJC-A003) in liver were determined by spectrophotometry using commercially available kits.

For the GSH assay, immediately after homogenization, an aliquot of homogenate was taken out to mix with trichloroacetic acid (20%, w/v), at the volume ratio of 10:1. This procedure has been confirmed to be able to precipitate all proteins in the homogenate and make GSH in the homogenate stable at 4°C for at least 2 h. The trichloroacetic acid-treated homogenate was centrifuged at 10,000 rpm and 4°C for 5 min. After centrifugation for 2 h, the supernatant was mixed with dinitrobenzoic aid (20 mg mL^-1^ in 0.2 M PBS, pH 8.0) and absorbance was read at 412 nm (Wang et al., 2008).

For antioxidant enzyme and other biomarkers assays, liver tissue was homogenized in ice cold 150 mM and pH 7.2 PBS containing 1 mM EDTANa_2_ (1:9, w/v), and then the homogenate was centrifuged at 15,000 rpm at 4°C for 15 min. Protein levels were determined by the Bradford dye-binding assay with BSA as the standard (Bradford, 1976). CAT activity was assayed on the basis of its ability to decompose H_2_O_2_ that was measured at 240 nm (Oberley et al., 1985). One unit of CAT activity was defined in terms of 1 nmol H_2_O_2_ consumed/min/mg protein. SOD activity was estimated by using the system of xanthine/xanthine oxidase and nitroblue tetrazolium. One unit of SOD activity was defined as the amount of protein that inhibits the rate of nitroblue tetrazolium reduction by 50% (Sun et al., 1988). Data are expressed as unit per milligram protein.

### Statistical Analysis

Data were presented as the mean ± SEM. The differences between groups were compared using ANOVA or Student’s *t*-test as appropriate using GraphPad Prism (GraphPad Software, Inc., La Jolla, CA, United States). *P*-value < 0.05, *P*-value < 0.01, and *P*-value < 0.001 were considered statistically significant.

## Results

### Effect of *C. gloeosporioides* Fermentation on Tea Infusion

The green tea infusion was bright yellow, while the *C. gloeosporioides-*contaminated tea infusion became dark brown (**Figure 1C**). *L*^∗^ value was reduced from 26.53 to 18.64, showing a decreased brightness, *a*^∗^ value was elevated from 0.66 to 4.55, showing an increased redness, and *b*^∗^ value was reduced from 6.05 to 4.36, showing a decreased yellowness.

After 48 h of fermentation, compared with green tea, the pH value, contents of tea polyphenols, caffeine, and L-theanine were decreased, respectively, by 12.7, 31.0, 26.2, and 8.3% in the *C. gloeosporioides*-contaminated tea infusion (**Figure 1D**). GC–MS analyses revealed that three new substances methoxy-phenyl-oxime, DIBP, and DBP were produced in *C*. *gloeosporioides*-contaminated tea compared with green tea infusion, with a retention time of 11.57, 50.869, and 54.01 min, respectively (**Figure 1E**).

### Changes of Lipid Metabolism in C57BL/6J Mice

**FIGURE 2:**
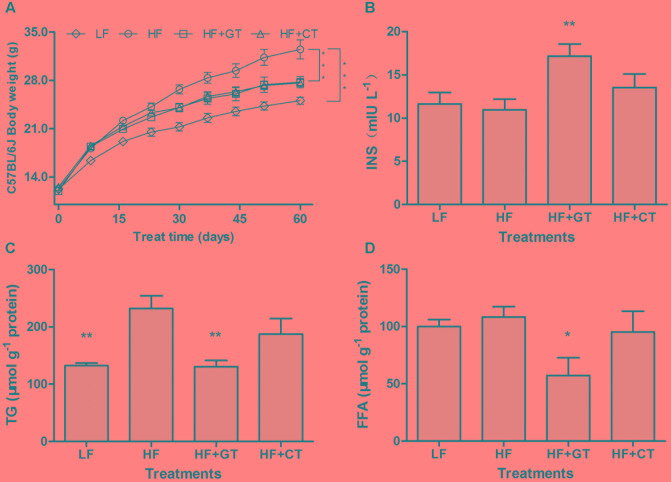
Changes of lipid metabolism in C57BL/6J mice. Body weight of C57BL/6J mice in different groups **(A)**. *C. gloeosporioides*-contaminated hindered the benefits of green tea on the increase of INS **(B)** and decrease of TG **(C)** and FFA **(D)**. Data were compared with HF group. Mean ± SEM. ^∗^*P* < 0.05; ^∗∗^*P* < 0.01; and ^∗∗∗^*P* < 0.001.

After being fed for 8 weeks, body weights of C57BL/6J mice in HF group were significantly increased compared with the mice in LF group (*P* < 0.001). But HF + GT and HF + CT groups were significantly decreased compared with HF group (*P* < 0.001, **Figure 2A**). There were no significantly differences between HF + GT and HF + CT groups.

Compared with HF group, drinking green tea (HF + GT group) can improve the obesity-induced insufficient INS (*P* < 0.01), but HF + CT group showed insignificant increase, which indicated that *C. gloeosporioides* contamination hinders the green tea’s biological effect of increasing INS secretion (**Figure 2B**). In addition, compared with HF group, the contents of hepatic TG (*P* < 0.01, **Figure 2C**) and FFA (*P* < 0.05, **Figure 2D**) in HF + GT group were significantly reduced. However, no lower level of the above indicators was detected in HF + CT group, suggesting that *C. gloeosporioides-*contaminated tea infusion hinders the lipid reduction function of green tea.

### Morphological and Functional Changes of Kidney, Liver, and Small Intestine in C57BL/6J Mice

**FIGURE 3:**
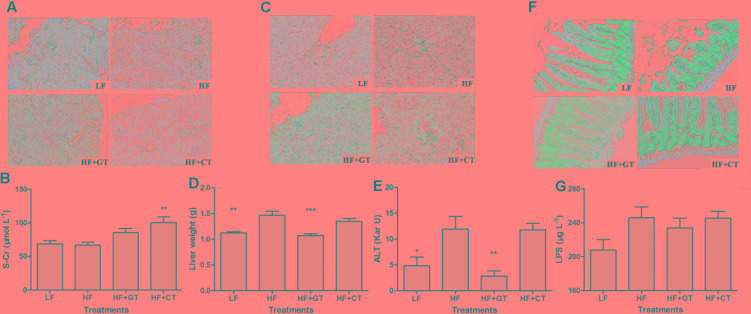
Morphological and functional changes of kidney, liver, and small intestine in C57BL/6J mice. Kidney tissues **(A)**, Liver **(C)**, and small intestine **(F)** by HE staining. S-Cr **(B)**, liver weight **(D)**, ALT **(E)**, and LPS **(G)** values in different groups. Data were compared with HF group. Mean ± SEM. ^∗^*P* < 0.05; ^∗∗^*P* < 0.01; ^∗∗∗^*P* < 0.001.

In LF group, the structure of renal glomeruli was normal, and the brush borders of renal tubular epithelium were integrity. In HF group, brush borders of renal tubular epithelium were detached, cells were swollen with a large amount of intracellular vacuoles, and part of renal tubular epithelium was detached and necrotized. In HF + GT group, the renal tubular epithelial cells were slightly swollen with a lot of intracellular vacuoles. In HF + CT group, the brush borders of the renal tubular epithelium were detached, cells were swollen with a large amount of intracellular vacuoles, and part of renal tubular epithelium were detached and necrotized, while some glomerulus were atrophic (**Figure 3A**). Meanwhile, compared with HF group, S-Cr value was significantly elevated in HF + CT group (*P* < 0.01, **Figure 3B**), suggesting that uptaking tea infusion contaminated with *C. gloeosporioides* damages the renal function in mice.

In terms of morphology, liver cells of LF group were clear and complete, structure of liver lobular was normal without steatosis. Liver cells of HF group showed mild hydropic degeneration with mild lymphocyte infiltration in the portal area. These symptoms were improved in HF + GT group, where cells were regularly and densely arranged with absence of hydroncus phenomenon. Meanwhile, cells in the HF + CT group still showed hydropic degeneration and mild lymphocytic infiltrate (**Figure 3C**). Then, compared with HF group, liver weight (*P <* 0.001, **Figure 3D**) and ALT (*P <* 0.01, **Figure 3E**) were significantly decreased in HF + GT group, but showed no significant difference in HF + CT group, indicating that contaminant of *C. gloeosporioides* hinders the protective effect of green tea on liver cells. Meanwhile, drinking contaminated tea infusion showed no significant impact on CAT, MDA, and SOD levels related to the redox capacity of liver in C57BL/6J mice (**Table 1**).

**Table 1:** Biochemical levels of C57BL/6J mice.

Treatments	Liver				Serum		
	CAT (U mg^-1^)	MDA (mmol g^-1^)	SOD (U mg^-1^)	GSH (mmol mg^-1^)	MCP-1 (ng L^-1^)	TNF-α (pg mL^-1^)	BUN (mmol L^-1^)
LF	0.30 ± 0.02^a^	0.40 ± 0.03^a^	3.14 ± 0.29^a^	53.72 ± 4.14^ab^	254.57 ± 33.97^a^	243.58 ± 23.51^b^	2.08 ± 0.56^a^
HF	0.19 ± 0.01^b^	0.42 ± 0.01^a^	2.78 ± 0.20^ab^	47.50 ± 2.18^bc^	193.91 ± 15.58^a^	309.08 ± 17.32^a^	2.21 ± 0.42^a^
HF + GT	0.18 ± 0.02^b^	0.46 ± 0.03^a^	2.80 ± 0.31^ab^	38.15 ± 4.63^c^	294.52 ± 42.64^a^	308.96 ± 19.94^a^	0.44 ± 0.06^b^
HF + CT	0.19 ± 0.04^b^	0.45 ± 0.04^a^	2.12 ± 0.24^b^	58.97 ± 2.97^a^	237.56 ± 40.94^a^	304.97 ± 17.58^a^	1.22 ± 0.32^ab^

*Data were compared with HF group. Mean ± SEM. Different letters indicated significance of the difference between two mean values at the *P* = 0.05 level. BUN, urea nitrogen; CAT, catalase; GSH, glutathione; MCP-1, monocyte chemotactic protein-1; MDA, malondialdehyde; SOD, superoxide dismutase; TNF-α, tumor necrosis factor-α.*

HE staining was utilized to observe intestinal villus in mice. The mice in the HF group showed losing and severe short villus, as well as swollen intestinal glands. These symptoms were improved in the HF + GT and HF + CT groups (**Figure 3F**). LPS content prompts the health level of intestinal flora in different hosts. In this study, serum LPS content of HF + GT group decreased compared with HF group, but these two groups showed no significant changes, indicating that drinking green tea and *C. gloeosporioides*-contaminated tea infusion were unlikely to cause dysfunction of flora in the digestive tract of mice (**Figure 3G**).

### Changes of Inflammation Factors in C57BL/6J Mice

Compared with HF group, the other three groups did not significantly change the contents of MCP-1 and TNF-α in serum (**Table 1**), indicating that drinking of tea infusion contaminated by *C. gloeosporioides* does not cause systemic inflammatory response in mice.

### Validation Tests with ICR Mice

**FIGURE 4:**
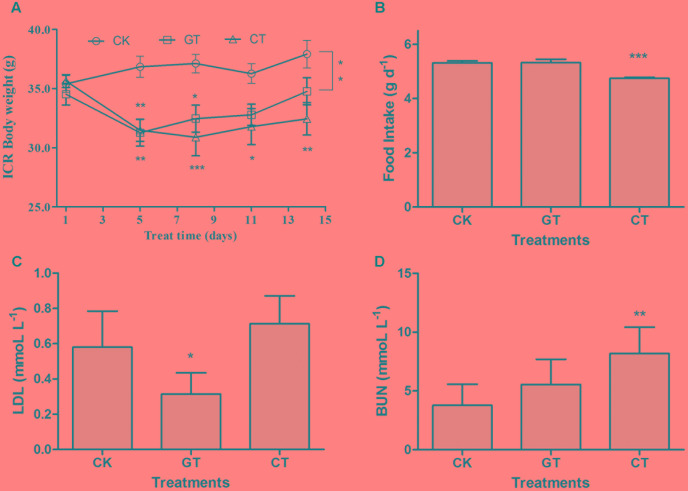
The influence of *C. gloeosporioides*-contaminated tea on ICR mice. Body weight **(A)**, food intake **(B)**, LDL **(C)**, and BUN **(D)** values in different groups. Data were compared with CK group. Mean ± SEM. ^∗^*P* < 0.05; ^∗∗^*P* < 0.01; and ^∗∗∗^*P* < 0.001.

In order to rule out the possibility that the effect of *C. gloeosporioides* contamination only happened in C57BL/6J mice, we tried to use another commonly used model to confirm our results. After being fed for 2 weeks, the body weights of ICR mice in both GT and CT groups were reduced compared with that in the CK group. The body weight increased by 7.11% in CK group and increased by 0.71% in GT group but decreased by 8.96% in CT group (**Figure 4A**). At 11–14 days, there was no significant difference observed in the body weight between GT and CK groups, while CT group showed a significant decrease compared with CK group (*P* < 0.01). Compared with CK group, food intake in CT group was significantly decreased (*P* < 0.001, **Figure 4B**). LDL content was significantly decreased (*P* < 0.05) in the ICR mice fed with green tea, but showed no decrease in ICR mice drinking *C. gloeosporioides-*contaminated tea infusion, which indicated that contamination of *C. gloeosporioides* hinders the original lipid reduction function of green tea (**Figure 4C**). Compared with the CK group, BUN value in CT group was significantly elevated (*P* < 0.01), while no significant increase was observed in mice of the GT group, suggesting that tea infusion contaminated with *C. gloeosporioides* damaged the renal function in ICR mice (**Figure 4D**). In addition, there were no differences between groups in TG, TC, HDL, ALT, AST, and S-Cr assays (**Table 2**).

**Table 2:** Biochemical levels of ICR mice.

Treatments	CK	GT	CT
TG (mmol L^-1^)	2.01 ± 0.16^ab^	1.87 ± 0.14^b^	2.47 ± 0.24^a^
TC (mmol L^-1^)	7.40 ± 0.66^a^	6.88 ± 0.31^a^	7.10 ± 0.48^a^
HDL (mmol L^-1^)	2.60 ± 0.15^a^	2.77 ± 0.16^a^	2.93 ± 0.12^a^
ALT (Kar U)	10.45 ± 3.93^a^	12.02 ± 3.87^a^	13.13 ± 3.98^a^
AST (Kar U)	24.46 ± 5.13^a^	27.81 ± 8.35^a^	25.28 ± 6.72^a^
S-Cr (μmol L^-1^)	355.1 ± 11.29^ab^	323.4 ± 8.939^b^	383.4 ± 15.03^a^

*Data were compared with CK group. Mean ± SEM. Different letters indicated significance of the difference between two mean values at the *P* = 0.05 level. ALT, alanine aminotransferase; AST, aspartate aminotransferase; HDL, high-density lipoprotein; S-Cr, serum creatinine; TC, total cholesterol; TG, triglycerides.*

## Discussion

The three major functional components of tea are tea polyphenols, caffeine, and L-theanine. A larger number of studies have confirmed that tea polyphenols has significant antioxidant effects (Forester and Lambert, 2011), and also exhibits functions in prevention and treatment of cancer and kidney diseases (Hisamura et al., 2006). Other functions included are anti- hypertensive, anti-inflammatory, hypolipidemic, strengthens INS activity (Anderson and Polansky, 2002). Caffeine, also known as 1,3,7-trimethylxanthine mainly acts as central nervous system stimulant (Nehlig et al., 1992). And it is demonstrated that the combination of catechins and caffeine induced inhibition of fat accumulation in mice through the improvement of hepatic lipid metabolism (Sugiura et al., 2012). L-Theanine, known as *N*-*ethyl*-γ-L-glutamine is a kind of unique free amino acid in tea leaves and has positive effects on allaying excitement (Kimura et al., 2007), and protecting the liver (Jiang et al., 2012). It is considered that green tea has beneficial effects against obesity, and the polyphenols or catechin in green tea demonstrates highest biological activity. In this study, three functional components (tea polyphenols, L-theanine, and caffeine) were significantly decreased when extracted green tea infusion was contaminated by *C. gloeosporioides.* Therefore, the functions of lipid-lowering, increasing INS secretion, and protecting liver greatly attenuated.

In this study, three new volatile components methoxy-phenyl-oxime, DIBP, and DBP were detected in tea infusion contaminated with *C. gloeosporioides* using GC–MS method. Methoxy-phenyl-oxime is a microbial fermentation product, which can be found in cheese (Jung et al., 2013) and haw juice (Zhao et al., 2015). DIBP and DBP have similar chemical properties, and belong to PAEs, which are colorless transparent liquids with slightly aromatic odor. PAEs are broad class fat-soluble compounds, commonly used as plasticizers, and are also detected in a variety of natural plant volatile oil (Li et al., 2008; Wang et al., 2009). They are widespread in human living environment, and can enter human body through breathing, diet, drinking water, and skin contact. They have been identified as fourth class of toxic chemicals, which can cause endocrinal and reproductive toxicity in humans (Kamrin, 2009; Martino-Andrade and Chahoud, 2010), and developmental toxicity in experimental animals (Fisher, 2004). In addition, epidemiological studies have shown that high exposure to phthalates can damage children’s intelligence and behavioral capacity (Cho et al., 2010), and also can cause asthma and allergic symptoms (Bornehag et al., 2004). In this study, DIBP or DBP was not detected in green tea. We speculated that they were present in the form of complexes with other components of tea infusion. However, whether the renal damage is related to PAEs, requires further investigation.

Endophytes are co-evolution products of microorganisms and plants, which are found in almost all the plants (Santoyo et al., 2016). On the one hand, some endophytes play active roles in plant growth and human usage (Tejesvi and Pirttilä, 2010; Waqas et al., 2013). On the other hand, some endophytes play negative roles (Kulkarni et al., 2007; Faeth et al., 2010), by producing toxic secondary metabolites and thus displaying serious impact on human food safety (Peraica et al., 1999; Ranaldi et al., 2009). In this study, the contaminated tea infusion had an adverse effect on mice, including that blocking green tea-induced decreasing levels of ALT, TG, FFA, LDL, and increased INS level compared with obese mice and increasing the BUN and S-Cr levels which was indicated that it induced kidney damage. Therefore, *C. gloeosporioides* control in tea plant cultivation should be reinforced. Picking of infected leaves should be avoided especially to produce tea concentrate and extractive such as caffeine and tea polyphenols with fresh leaves as raw materials. Enough attention should be paid to establishing convenient, rapid and economical methods to test fresh tea leaves.

## Conclusion

Contamination with tea plant *C. gloeosporioides* pathogenic fungi caused brown stain in the green tea infusion, and produced toxic compound such as DIBP and DBP. And three main functional components of tea (tea polyphenols, caffeine and L-theanine) were lowered, respectively. The animal experiments revealed that *C. gloeosporioides*-contamination tended to damage the renal function (elevated S-Cr and BUN) in mice, and to hinder the original functions of green tea, such as protecting liver (lowered ALT), regulating lipid metabolism (decreased LDL and FFA) as well as modulating glucose metabolism (increased INS). Increased attention should be paid to the safety of tea processing in order to establish specific rules of supervisory control.

## Ethics Statement

This study was carried out in accordance with the recommendations of the guidelines of American Association for Accreditation of Laboratory Animal Care. The protocol was approved by the Animal Use and Care Committee of Nanjing Agricultural University.

## Author Contributions

JL and KS finished major experiments and wrote the main manuscript text. JL, KS, QM, and LW prepared **Figures 1–4**. JC revised the manuscript. XC and XL designed all experiments and revised the manuscript. All authors reviewed the manuscript.

## Conflict of Interest Statement

The authors declare that the research was conducted in the absence of any commercial or financial relationships that could be construed as a potential conflict of interest.

## Footnotes

1 https://blast.ncbi.nlm.nih.gov/Blast.cgi

## Abbreviations

ALT: alanine aminotransferase
AST: aspartate aminotransferase
BUN: blood urea nitrogen
*C. gloeosporioide*s: *Colletotrichum gloeosporioides*
CAT: catalase
DBP: dibutyl phthalate
DIBP: diisobutyl phthalate
FBG: fasting blood sugar
FFA: free fatty acid
GSH: glutathione
HDL: high-density lipoprotein
HPLC: high-performance liquid chromatography
INS: insulin
ITS: internal transcribed spacer
LDL: low-density lipoprotein
LPS: lipopolysaccharide
MCP-1: monocyte chemotactic protein-1
MDA: malondialdehyde
PAEs: phthalic acid esters
S-Cr: serum creatinine
SOD: superoxide dismutase
TC: total cholesterol
TG: triglycerides
TNF-α: tumor necrosis factor-α

## Chemical Compounds

Caffeine (PubChem CID: 2519); L-theanine (PubChem CID: 439378); Methoxy-phenyl-Oxime (PubChem CID: 9602988); diisobutyl phthalate (PubChem CID: 6782); dibutyl phthalate (PubChem CID: 3026).
